# Impact of Shutdown due to COVID-19 Pandemic on Aerosol Characteristics in Kanpur, India

**DOI:** 10.5696/2156-9614-10.28.201201

**Published:** 2020-11-19

**Authors:** Nidhi Shukla, Gautam Kumar Sharma, Parinita Baruah, V. K. Shukla, Prashant Gargava

**Affiliations:** Central Pollution Control Board, Parivesh Bhawan, East Arjun Nagar, Delhi, India

**Keywords:** COVID-19, Kanpur, aerosol, AERONET, air quality

## Abstract

**Background.:**

Since March 2020, the number of confirmed COVID-19 positive cases have steadily risen in India. Various preventive measures have been taken to contain the spread of COVID-19. With restrictions on human activities, anthropogenic emissions driving air pollution levels have seen a reduction since March 23, 2020, when the government imposed the first nationwide shutdown. The landlocked Indo-Gangetic Plain (IGP) has many densely-populated cities, witnessing high levels of particulate matter due to both nature-driven and anthropogenic elements. Kanpur is an urban metropolis in the IGP with high aerosol loading, and this paper explores the impact of restricted anthropogenic activities on aerosol characteristics in Kanpur.

**Objectives.:**

This study aims to investigate the change in aerosol optical depth level and its related parameters during the shutdown phases in Kanpur city compared to the same time periods in 2017–2019.

**Methods.:**

Aerosol optical properties such as aerosol optical depth (AOD) at 500 nm, Angstrom exponent (AE), fine mode fraction (FMF) of AOD at 500 nm and single scattering albedo (SSA) at 440 nm were obtained from the Aerosol Robotic Network (AERONET) station operating in Kanpur from the 1st March to the 30th April for 2017–2020.

**Results.:**

A significant decrease in aerosol loading was observed during the shutdown period compared to the pre-and partial shutdown periods in 2020 as well as during the same time periods of 2017–2019. Mean AOD, FMF and SSA were 0.37, 0.43 and 0.89, respectively, during the shutdown period in 2020. A 20–35% reduction in mean AOD levels was observed during the shutdown period in 2020 as compared to the same period in 2017–2019.

**Conclusions.:**

The shutdown led to an improvement in air quality due to decreases in anthropogenic emissions. As fine particles, typically from urban and industrial emissions, dominate episodic air pollution events, this study can be further utilized by the scientific community and regulators to strengthen the emergency response action plan to check high pollution episodes in Kanpur city until cleaner technologies are in place.

**Competing Interests.:**

The authors declare no completing financial interests.

## Introduction

India has one of the highest exposure levels to air pollution globally.^1^ The effects of air pollution on health are well known, with the International Agency for Research on Cancer (IARC) classifying outdoor air pollution as a carcinogen.^2^ There has been an increasing trend of carbon-containing aerosols and precursor emissions in southern Asia owing to the increasing population associated with industrial development.^3^

As per the World Health Organization's (WHO) Global Ambient Air Quality Database, 14 of the top 20 most polluted cities in the world are located in India.^4^ Furthermore, a quick analysis of the Central Pollution Control Board (CPCB) ambient air quality monitoring data for 2018 revealed that for most major cities, especially in northern India, annual particulate matter of 10 micrometers or less (PM_10_) averages more than the annual standard of 60 μg/m^3^ established by the National Ambient Air Quality Standards (NAAQS).^5^

The Indo-Gangetic Plain (IGP), which covers most parts of northern India, is a major centre of agriculture, commercial and industrial activities, and has the highest aerosol loading in India.^6,7^

Air quality issues in the IGP revolve around high levels of particulate matter (PM_10_ and particulate matter of 2.5 micrometers or less (PM_2.5_)) due to aerosols from alluvium plains and anthropogenic activities in several million plus cities located in this belt. The IGP is landlocked and surrounded by the Himalayas in the north and the Thar desert in the west. It stays polluted particularly during the winter season when pollution levels tend to go up on account of increased emissions from local sources, biomass burning, including paddy stubble, festivals and unfavorable meteorological conditions resulting in poor dispersion of pollutants.^8^ It has also been suggested that from 2001–2010, anthropogenic emissions over the IGP have continued to increase mainly due to fossil-fuel and biomass combustion.^9^

As per a study by the Energy Policy Institute at the University of Chicago, from 1998–2016, the particulate pollution levels in IGP were about twice as high as the rest of the country.^10^ Sustained exposure to particulate pollution reduced the average lifespan of residents by 3.7 years in 1998 and by 3.4–7.1 years further in 2016.^10^ Moreover, the emissions from the IGP contributed to around 46% of the estimated 1.1 million annual premature deaths from PM_2.5_ in India.^11^ Control of anthropogenic emissions has been discussed and evaluated in several studies.^12–15^

To tackle rising air pollution, the CPCB has identified 122 cities which exceed the prescribed NAAQS, based on air quality data from 2014–2018, and has termed them as non-attainment cities. City-specific action plans targeting major emission sources specific to each city are being prepared and implemented. Major components of these air quality management plans are short (to be completed within 6 months), medium (to be completed within 1 to 3 years) and long term (to be completed in three years or more) actions. An important component of an air quality management plan is the development of an emergency response system. While various source-specific actions aim for systemic control of emissions, emergency response systems such as the Graded Response Action Plan in Delhi may help to prevent episodic high air pollution events during critical air quality conditions. Critical conditions mainly arise due to unfavorable meteorology and high emissions due to festivals. Recently, the Ministry of Environment, Forest and Climate Change, Government of India, has launched the National Clean Air Programme as a national level implementation strategy for pan-India to reduce air pollution levels by 20 to 30% in terms of PM_10_ and PM_2.5_ concentrations by 2024.^16^ The target set by the National Clean Air Programme is significant as it has been estimated that a 25% reduction in PM_2.5_ will translate to a national average life expectancy increase of 1.3 years with 2016 levels as the baseline.^17^

**Abbreviations**
*AE*: Angstrom exponent
*AERONET*: Aerosol Robotic Network
*AOD*: Aerosol optical depth
*CAAQM*: Continuous Ambient Air Quality Monitoring
*CPCB*: Central Pollution Control Board
*FMF*: Fine mode fraction
*IARC*: International Agency for Research on Cancer
*IGP*: Indo-Gangetic Plain
*IIT*: Indian Institute of Technology
*NAAQS*: National Ambient Air Quality Standards
*RH*: Relative humidity
*SSA*: Single scattering albedo
*WHO*: World Health Organization

The ongoing COVID-19 pandemic has affected more than 216 countries/areas/territories.^18^ Many countries have responded to the pandemic by implementing a lockdown and imposing restrictions like social distancing and promotion of isolation measures. With a lockdown in place due to the outbreak of the COVID-19 pandemic in numerous countries, a considerable improvement in air quality has been reported from major cities in the world.^19–21^

In India, from January 30 to May 27, 2020, around 151,767 confirmed cases of COVID-19 were reported with 4,337 deaths.^22^ To minimize exposure and to combat the ongoing outbreak of COVID-19, the Government of India announced a nationwide shutdown from March 25, 2020 to April 14, 2020. However, the shutdown was further extended until May 3, 2020 with some relaxations from April 20, 2020.^23–25^ Under the lockdown, stringent travel restrictions were in place along with the shutdown of non-essential activities which included air polluting sectors.

In the present study, aerosol data were obtained from the Aerosol Robotic Network (AERONET) site located at Kanpur to understand the role of anthropogenic emissions and their absence during the shutdown period.

Aerosol data before and after the complete shutdown as well as during the same time periods of previous years (2017–2019) were compared. The analysis is supplemented by data obtained from the Continuous Ambient Air Quality Monitoring (CAAQM) station in Kanpur operated by the Uttar Pradesh Pollution Control Board.

## Methods

Kanpur is located at 26.449923° N, 80.331874° E, in the state of Uttar Pradesh in India. As per results of the 2011 census, Kanpur (municipal corporation + outgrowth) has a population of 2,768,057.^26^

Kanpur is a major commercial and industrial center located in the IGP. As per the WHO Ambient Air Quality Database 2018, Kanpur was ranked as the most polluted city in the world in terms of annual mean PM_2.5_ concentrations.^4^

Kanpur has a considerable presence of industries, notably textile and leather processing industries.^27^ A CPCB source apportionment study identified particulate pollution as the main concern in Kanpur where levels of suspended particulate matter, PM_10_ and PM_2.5_ were 2.5–3.5 times higher than the acceptable levels. Vehicles, road dust and domestic fuel burning were the most prominent sources for PM_10_, while open burning (7–23%), road dust (3–6%), coal (0–13%), vehicles (28–37%) and secondary particles (15–30%) were major contributors to PM_2.5._^28,29^ Considering the high levels of particulate matter in Kanpur, it was also identified as a non-attainment city by the CPCB.

Data from the ground-based observation (AERONET) station in Kanpur were used in the present study. The station is located at 26.51278° N, 80.23164° E in the Indian Institute of Technology (IIT) Kanpur campus.

**Figure 1:**
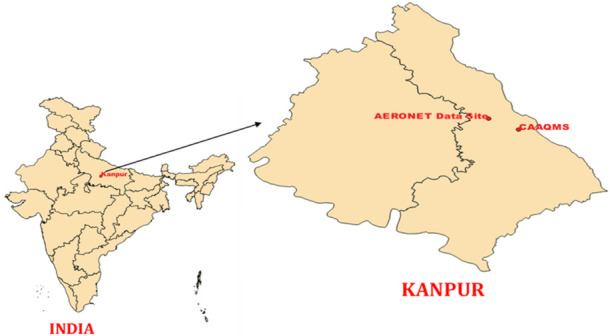
Representation of data sites in Kanpur

Meteorological and air quality data (air quality index) from a CAAQM station operated by the Uttar Pradesh Pollution Control Board in Kanpur were also used. The CAAQM station is located at 26°28′13.1″ N, 80°19′22.8″ E in Nehru Nagar, Kanpur.^30^ Figure 1 depicts the location of Kanpur and the sites from where data has been obtained.

### Data collection methods

The AERONET is a network of ground-based sun photometers that measure atmospheric aerosol properties. Developed by the National Aeronautics and Space Administration (NASA) under the leadership of Dr. Brent Holben, AERONET provides ground-based calibrated data for NASA and other international satellite missions. Data is automatically cloud-cleared and quality-assured with pre-field and post-field calibration. The AERONET data is also available at three different levels, level 1.0 (raw or unscreened data), level 1.5 (cloud-screened data) and level 2.0 (cloud-screened and quality assured data).^31^

Atmospheric aerosols are solid or liquid particles suspended in air and may be formed due to natural or anthropogenic emissions. The Aerosol Robotic Network provides aerosol optical depth (AOD), Angstrom exponent (AE), fine mode fraction (FMF), single scattering albedo (SSA), refractive indices, aerosol size distribution and other products which can represent the absorptivity, aerosol volume distribution, scattering direction, fine particle ratio, and other aerosol optical and physical properties.^32^

Single scattering albedo is the ratio of the scattering coefficient to the extinction coefficient of the total aerosol column and affects radiative transfer. While FMF indicates the contribution of fine particles, SSA at 440 nm distinguishes between absorbing and non-absorbing aerosols. Consequently, depending on FMF and SSA, the aerosol types are classified as highly-absorbing, moderately-absorbing, slightly-absorbing, mixed, dust, and scattering.^33^ Several studies have used AERONET products to determine the class of aerosols for different areas and seasons.^34–36^

**Figure 2:**
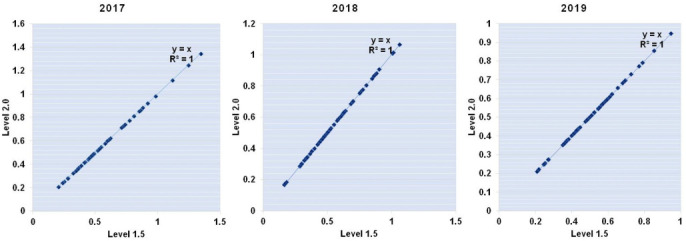
Scatter plot presenting correlation between Level 1.5 and Level 2.0 AOD for 2017, 2018 and 2019

In this study, AOD, AE, FMF and SSA data (level 1.5) from 2017–2020 were obtained from the AERONET Kanpur site. Although level 2.0 data is considered more accurate than level 1.5, level 2.0 data points were not available for 2020. However, there is strong correlation between level 1.5 and level 2.0 AOD for 2017, 2018 and 2019, as depicted in scatter plots in Figure 2.

## Results

**Figure 3:**
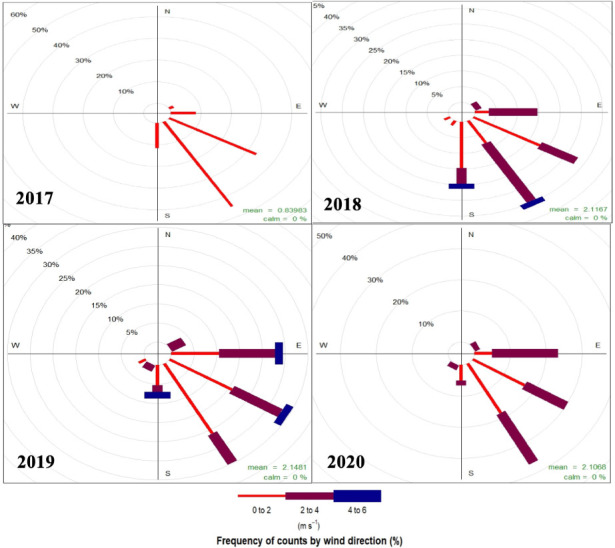
Windrose for 1st March–30th April of 2017–2020

Meteorological properties at Kanpur were compared for the study period (March 1^st^ to April 30^th^) during 2017–20. Figure 3 shows the wind rose for the study period during 2017–2020. Dominant winds over the years were from the east and south-east directions, with average wind speed of 2.1 m/s for 2018, 2019 and 2020 and 0.8 m/s for 2017.

Relative humidity (RH) and temperature are compared in Table 1 for the study period of 2017–2020. Relative humidity ranged from 45–87% with a mean of 64% in 2020, while average RH was 58%, 55% and 54% in 2019, 2018 and 2017, respectively. Ambient temperature was similar for 2019 and 2020 and ranged between 32–44°C whereas it varied between 24–32°C for 2017 and 2018.

**Table 1:** Comparative Temperature and Relative Humidity for the Study Period of 2017–2020

Parameter	Minimum	Maximum	Mean	SD
Temp-20	33.69	44.14	36.33	2.65
Temp-19	31.94	44.18	35.18	2.41
Temp-18	26.31	31.07	28.24	1.25
Temp-17	24.48	32.67	29.54	1.93
RH-20	45.57	86.79	64.27	11.66
RH-19	40.13	84.76	57.88	8.87
RH-18	39.88	69.45	55.08	7.45
RH-17	35.86	77.51	54.34	7.97

### Aerosol optical depth and Angstrom exponent

**Figure 4:**
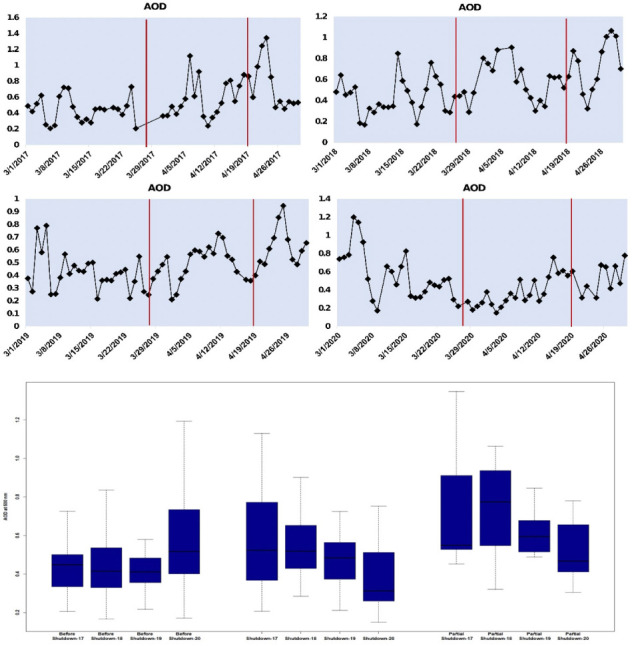
Variation of AOD values observed before shutdown (March 1st–24th), shutdown (March 25th–April 19th) and the partial shutdown (April 20th–30th) periods for 2017–2020

Aerosol optical depth and the AE at 500 nm, from March 1st to April 30th during 2017–2020 are compared in this section. Figure 4 depicts the variation of the AOD values observed before the shutdown (March 1st–24th), during the shutdown (March 25th–April 19th) and the partial shutdown (April 20th–30th) periods for 2017–2020.

Although the term shutdown is only applicable to the said period in 2020, for simplicity, the corresponding time periods in previous years will also be called by these same names but differentiated by year. A significant drop in aerosol loading was identified during the COVID-19 shutdown with mean AOD at 500 nm for March 25th–April 19th 2020 at 0.37 which is the lowest as compared to the mean of the past three years for the same period and when compared with the values observed before the shutdown and during the partial shutdown periods. A 20–35% reduction in mean AOD levels was observed during the shutdown period in 2020 compared to the same period in 2017–2019.

**Figure 5:**
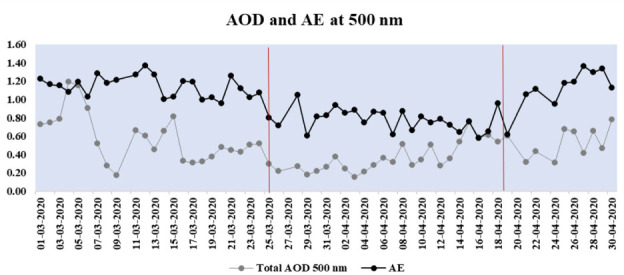
Time series of daily aerosol optical depth values at 500 nm Angstrom exponent

Figure 5 shows the time series of mean daily AOD values at 500 nm and AE, measured over Kanpur from March 1, 2020 to April 30, 2020.

During the shutdown period (2020), the Angstrom wavelength exponent ranged from 0.57–1.05 and mean AE at 500 nm was observed to be 0.77.

In addition, statistical significance was checked by applying the P value, i.e. P < 0.05 for a statistically significant difference at the 95% confidence level. P values for comparisons between AOD during the shutdown period and AOD for the same period in 2017, 2018 and 2019 were 0.001, 0.003 and 0.012, respectively. P values for the correlation between the before shutdown and the shutdown period and between the shutdown period and partial shutdown period for 2020 were 0.01 and 0.004, respectively.

### Fine mode fraction and single scattering albedo

**Figure 6:**
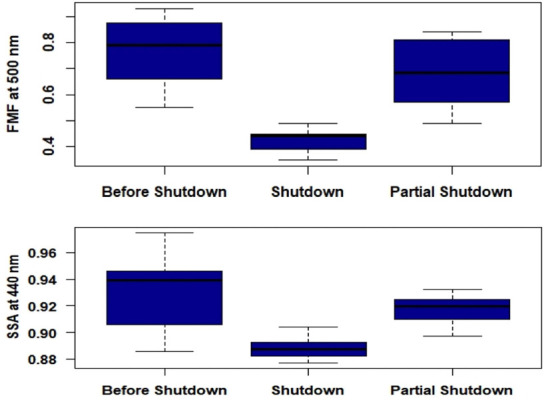
Comparative analysis for before shutdown, shutdown and partial shutdown in 2020 of fine mode fraction (FMF) and single scattering albedo (SSA) at 440 nm

Fine mode fraction during the shutdown was lowest (0.43) in 2020 in comparison to the before shutdown and partial shutdown periods (*Figure 6*).

### Nature of aerosol during episodes

The air quality index transforms complex air quality data of various pollutants into a single number (index value), nomenclature and color. There are six air quality index categories: good, satisfactory, moderately polluted, poor, very poor, and severe, decided on the basis of ambient concentration values of air pollutants and their likely health impacts (known as health breakpoints).^37^ The air quality index of the winter months of Kanpur for 2017–19 published by the CPCB were analyzed. The maximum number of ‘severe' air quality days were observed during November and December, 2017. It was also noted that during the same period (November, 2017) most cities in IGP from Ludhiana to Muzaffarpur were experiencing very poor to severe air quality. Air quality levels were in the severe category for 165 hours in the Delhi National Capital Region during this period.^38^

Aerosol characteristic and aerosol loading of an episodic event (high pollution event) like November 2017 were observed. Table 2 summarizes the optical properties of aerosol during the episodic pollution event of November 2017. The AOD ranged from 0.57–2.04 and fine mode AOD ranged from 0.52–2.00 during this high pollution event.

**Table 2:** Descriptive Statistics of AOD, Fine Mode & Coarse Mode AOD and Fine Mode Fraction during Episodic High Pollution Event of November 2017

	**AOD**	**Fine Mode AOD**	**Coarse Mode AOD**	**FMF**
**Min**	0.57	0.52	0.02	0.87
**Max**	2.04	2.00	0.09	0.98
**Mean**	1.12	1.08	0.04	0.96
**Median**	1.06	1.02	0.04	0.97

Abbreviations: AOD, aerosol optical depth; FMF, fine mode fraction

## Discussion

Wind speed and wind direction during the study period were observed to be similar from 2017–2020 while the RH and temperature were higher in 2020 as compared to previous years. As per previous studies for aerosol properties over China, when RH was greater than 60%, significant changes in light scattering aerosols occurred due to hygroscopic growth.^39–41^

Although greater than 60% RH was observed during the study period of 2020, lower aerosol loading suggests the impact of the reduction in emission sources due to the shutdown.

Analysis of AERONET data for Kanpur from 2001–2008 in a previous study observed that aerosol loading (AOD) at 500 nm was more than 0.4 throughout the year and reached its maximum value in May and June, coinciding with a peak in dust generation activity. As per the study, a consistent increase from March to June was also observed in the monthly variation of AOD.^42^ Such conditions prevailed during the study period in 2017–19 as well. However, in 2020, aerosol loading was noticeably different, possibly due to the implementation of the shutdown in April.

It appears that coarse mode aerosols were dominant during the shutdown period due to the reduction in anthropogenic sources during this period. This may be corroborated as negative AE values were observed during the shutdown period. AE ≤ 1 indicates coarse-mode aerosols of effective radius usually greater than 0.5 μm, primarily due to dust outflows or sea spray and AE ≥ 1 usually indicates fine-mode aerosols of effective radius smaller than 0.5 μm, typically associated with biomass burning and urban pollution.^39,43^ Positive AE values are associated with an increase in fine anthropogenic emissions, whereas negative AE values suggest an increase in natural coarse-mode aerosols.^9^

Fine mode fraction can also be used to distinguish between the dominance of fine or coarse particles in an aerosol distribution, with fine particles typically associated with FMF > 0.6 and coarse particles with FMF < 0.4. Mixed aerosol distribution with both coarse and fine particle aerosols is indicated when FMF lies between 0.4 and 0.6.

The fine mode aerosols over urban, industrialized and densely populated regions are mainly due to gas to particle conversion mechanism-produced aerosols (fossil fuel, biomass combustion; i.e. anthropogenic), while coarse mode aerosols such as windblown mineral dust and sea salt particles mainly arise from natural sources.^44^

Single scattering albedo threshold for scattering and absorbing aerosols has been noted to be 0.95. Single scattering albedo at 440 nm between 0.9–0.98 indicate urban/industrial aerosol, 0.92–0.93 indicates desert dust and 0.89–0.95 indicates biomass burning.^34,35^

The aerosols over Kanpur in the pre-and partial shutdown periods seem to be associated with black carbon-based slightly absorbing aerosol with higher contribution of fine particles, while during the shutdown period appears to be of mixed nature i.e., a mixture of coarse and fine particle aerosols.^31^ While coarse mode particles are found to contribute a high percentage to the total aerosol content during summer and pre-monsoon season in Kanpur, there was certainly a reduction in fine aerosols.^34, 45–47^ This is also consistent with a reduction in FMF, possibly due to the reduced anthropogenic emissions as a result of the shutdown. Several studies have indicated that AOD during post-monsoon and winter seasons can be partly attributed to coal fired thermal power plants, biomass burning, combustion of unclean fuels along with industrial and vehicular emissions, among others.^45,47–49^

### Importance of source control during episodes

Previous studies have indicated a distinct seasonal variation in aerosol optical properties in the IGP. This is mainly due to various emission sources, meteorological and atmospheric variations, with the aerosol loading primarily of anthropogenic origin during winters, with some studies designating over 70% contribution by fine mode urban/industrial aerosols. On the other hand, coarse mode aerosols, possibly dust due to long range transport from western arid regions, contribute prominently to AOD during the summer.^7,45,46,50–55^

A study of severe air quality events in Kanpur reaffirmed that episodic days were associated with enhanced presence of fine anthropogenic aerosols and/or biomass burning in post-monsoon and winter seasons.^45^ Fine-mode aerosols tend to originate from burning of bio-fuels and biomass along with fossil-fuel combustion in vehicles, industries and power plants.^42,49,52,56,57^ Moreover, there is less inter-annual variability in fine-mode aerosol emission rates except for slight variations in the quantum of biomass burning and fuel consumption during the winter.^42^

The shutdown due to the COVID-19 pandemic and subsequent improvement in air quality suggests that control of anthropogenic sources of air pollution will yield benefits, highlighting the importance of an emergency response action plan restricting emission sources during high pollution events for cities across India.

While the present study only highlighted the improvement in air quality during the shutdown period, the impact of a shutdown as an emergency measure during episodic events needs to be quantitatively assessed, with consideration given to the practicality of shutdown measures and inter-seasonal variations. Further studies are needed to facilitate implementation of effective measures for episodic events with a detailed analysis of the variation in air quality with attention to the impact of meteorology.

## Conclusions

The present study examined variations in aerosol optical properties at the Kanpur AERONET site during the shutdown due to the COVID-19 pandemic in India. Due to restricted anthropogenic activities during the shutdown period, fine mode aerosols were considerably reduced and coarse-mode particles were prominent during the shutdown, while the pre-shutdown and partial shutdown periods were characterized by fine particles with slightly absorbing nature. As fine particles are observed to be dominant during episodic high pollution events when pollution levels increase due to local and regional emissions coupled with adverse meteorology, restriction on anthropogenic activities may be planned with due consideration given to the air quality improvement observed during the shutdown phases. City-level air quality management plans could be strengthened based on the improvement in air quality achieved due to various restrictions during the COVID-19 shutdown. An emergency response action plan with graded restrictions on polluting activities can be used as a preliminary air quality management tool by regulators for controlling anthropogenic emissions during critical air quality periods.
